# Polyphenols increase circulating lipids but improve LDL particle quality and reduce LDL oxidation in postmenopausal women: metabotype- and age-dependent effects in a randomised, placebo-controlled crossover trial

**DOI:** 10.1007/s00394-026-04027-2

**Published:** 2026-06-29

**Authors:** María García-Nicolás, María Paula Jarrín-Orozco, María Romo-Vaquero, Concepción Carrascosa, Francisco Avilés-Plaza, Miriam Martínez-Villanueva, José Antonio Noguera, María Ángeles Ávila-Gálvez, Juan Carlos Espín

**Affiliations:** 1 Laboratory of Food & Health, Research Group on Quality, Safety, and Bioactivity of Plant Foods, CEBAS-CSIC, 30100 Campus de Espinardo, Murcia, Spain; 2 Department of Obstetrics and Gynaecology, Virgen de La Arrixaca University Hospital, El Palmar, 30120 Murcia, Spain; 3 Institute of Biomedical Research (IMIB-Arrixaca), Hospital Clínico Universitario Virgen de la Arrixaca, 30120 Murcia, Spain

**Keywords:** Cardiovascular risk, Apolipoprotein B, Oxidised LDL, Polyphenols, Menopause, Metabotype

## Abstract

**Purpose:**

Menopause increases cardiometabolic risk via dyslipidaemia, oxidative stress, and low-grade inflammation. Polyphenols may modulate these processes, but human evidence is inconsistent. We investigated the effects of polyphenol supplementation on lipid profile, LDL oxidation, and polyphenol-related gut microbiota metabotypes in postmenopausal women.

**Methods:**

In the PolyPAUSE randomised, placebo-controlled, crossover trial, 90 mildly hypercholesterolemic, non-medicated postmenopausal women received a daily polyphenol mixture providing ellagitannins+ellagic acid (312.0 ± 30.9 mg), resveratrol (133.2 ± 10.1 mg), and isoflavones (166.3 ± 27.3 mg) for 8 weeks, followed by a 4-week washout and an 8-week placebo period. Serobiochemical and bone-related markers were analysed at hospital laboratories. Apolipoprotein B (ApoB), oxidised LDL (oxLDL), and lipopolysaccharide-binding protein (LBP) were measured by ELISA, and myeloperoxidase activity by spectrophotometry. Gut microbiota metabotypes were classified as urolithin A/B (UMA/UMB), equol producers/non‑producers (EP/ENP), and lunularin producers/non‑producers (LP/LNP) using UPLC‑ESI‑QTOF‑MS.

**Results:**

Polyphenol supplementation did not affect LBP or bone markers. Total cholesterol (Tchol), LDL-cholesterol (LDLc), and triglycerides (TGs) increased by 7%, 9.5%, and 16%, respectively (*p* < 0.001) in ~ 80% of completers (*n* = 78), with age‑related increases. ApoB showed a borderline reduction (*p* = 0.056), and oxLDL decreased (*p* < 0.001). Ratios indicating LDL particle quality and oxidative burden (LDLc/ApoB, oxLDL/LDLc, oxLDL/ApoB) improved (*p* < 0.001) despite higher circulating lipid concentrations. Myeloperoxidase activity showed marginal decreases in the full cohort (*p* = 0.08) and in LNP (*p* = 0.06). Metabotyping showed that the strongest oxLDL reduction occurred in EP (65%, *p* < 0.001) and the UMA + EP+LNP cluster (60%, *p* < 0.001).

**Conclusion:**

Polyphenol supplementation increased Tchol, LDLc, and TGs age-dependently, yet improved LDL oxidative quality. The extent of oxLDL reduction depended on metabotypes, supporting precision health approaches to better characterise cardiometabolic responses to polyphenol intake in postmenopausal women.

**Supplementary Information:**

The online version contains supplementary material available at 10.1007/s00394-026-04027-2.

## Introduction

Menopause, typically occurring at 50–52 years, is characterised by ovarian follicle depletion and estrogen decline, leading to vasomotor, metabolic, and cardiovascular alterations, with over 80% of women experiencing symptoms [1].

The decline in estrogen levels increases cardiovascular risk by disrupting energy balance and promoting endothelial dysfunction, hypertension, and dyslipidaemia [2, 3]. Although estrogen deficiency is a central driver, oxidative stress, altered lipoprotein metabolism, and low-grade inflammation also contribute to the heightened cardiovascular risk during menopause [4].

Polyphenols are plant-derived compounds with the potential to reduce the risk of chronic diseases. Experimental and clinical studies have linked polyphenol intake to lower oxidative and inflammatory stress, improved lipid regulation, and enhanced mitochondrial function [5–8]. Polyphenols may benefit cardiovascular risk and lipid oxidation, although effects on circulating lipids remain uncertain. Interindividual variability in polyphenol metabolism likely contributes to inconsistent results [9].

In postmenopausal women, polyphenols may help reduce lipid alterations, oxidative stress, and inflammation, although evidence remains heterogeneous. Isoflavones are among the most extensively studied polyphenols in postmenopause. Meta‑analyses report modest effects of isoflavones on TGs and HDLc, with no consistent impact on LDLc or Tchol [10, 11]. Evidence for other polyphenol classes is likewise variable. Grape- and olive-derived polyphenols have been shown to improve serum lipids in postmenopausal women [12, 13]. However, long-term resveratrol supplementation did not alter Tchol, LDLc, HDLc, or TGs in a 12-month intervention [14]. Recent systematic reviews confirm the limited and variable impact of resveratrol on lipid markers, highlighting the need to determine optimal dosing strategies and responder profiles [15, 16].

A significant source of variability in polyphenol responses, beyond their structural diversity, is their extensive metabolism by the gut microbiota, producing metabolites with distinct bioavailability and biological effects. Among the best-characterised examples, equol, derived from isoflavones, and urolithin A, derived from ellagic acid, display antioxidant, anti-inflammatory, and cardioprotective properties [17]. Interindividual differences in the production of these metabolites have led to the classification of gut microbiota metabotypes related to polyphenol metabolism. Individuals differ in their capacity to produce equol, urolithins, or other bioactive metabolites, and combinations of metabotypes can coexist in the same individual (metabotype clusters), resulting in diverse biological potential [18–21].

We hypothesised that high‑dose supplementation with a mixture of pomegranate, red clover and *Polygonum cuspidatum* extracts (rich in the polyphenols ellagic acid, isoflavones and resveratrol, respectively) could influence blood lipid levels in postmenopausal women. Given the well‑known interindividual variability in polyphenol metabolism, we further hypothesised that gut microbiota metabotypes could modulate the response to this supplementation.

## Materials and methods

### Chemicals and reagents

High-performance liquid chromatography (HPLC)-grade solvents, including acetonitrile, dimethyl sulfoxide (DMSO), methanol, and formic acid, were obtained from JT Baker (Deventer, The Netherlands). The polyphenols and gut microbial standards are detailed in the Supplementary Information.

### Subjects and study design

This randomised, double‑masked, placebo‑controlled, two‑period crossover Phase‑II trial (20 weeks) was conducted within the PolyPAUSE Project. The full study design, recruitment procedures, and metabotyping protocol have been described previously [20, 21]. The protocol was approved by the Ethics Committees of CSIC (ref. 249/2023), IMDEA‑Food (ref. PI‑065), and Virgen de la Arrixaca University Hospital (ref. 2023‑4‑9‑HCUVA). All participants provided written informed consent in accordance with the Declaration of Helsinki and its amendments. The trial was registered at ClinicalTrials.gov (NCT07182370). Participants were recruited between September 2023 and January 2025.

Postmenopausal women (≥ 1 year without menstruation), aged 45–59 years and BMI > 18 kg/m², were recruited at CEBAS‑CSIC (Murcia), IMDEA‑Food (Madrid), and Virgen de la Arrixaca Hospital (Murcia). Women using medication, smokers, vegetarians, or with allergies were excluded (full criteria in [20]).

Participants were metabotyped for ellagitannin, isoflavone, and resveratrol metabolism following a standardised short‑term polyphenol intake, as previously reported [20]. Baseline assessments were performed before any supplementation.

**Fig. 1:**
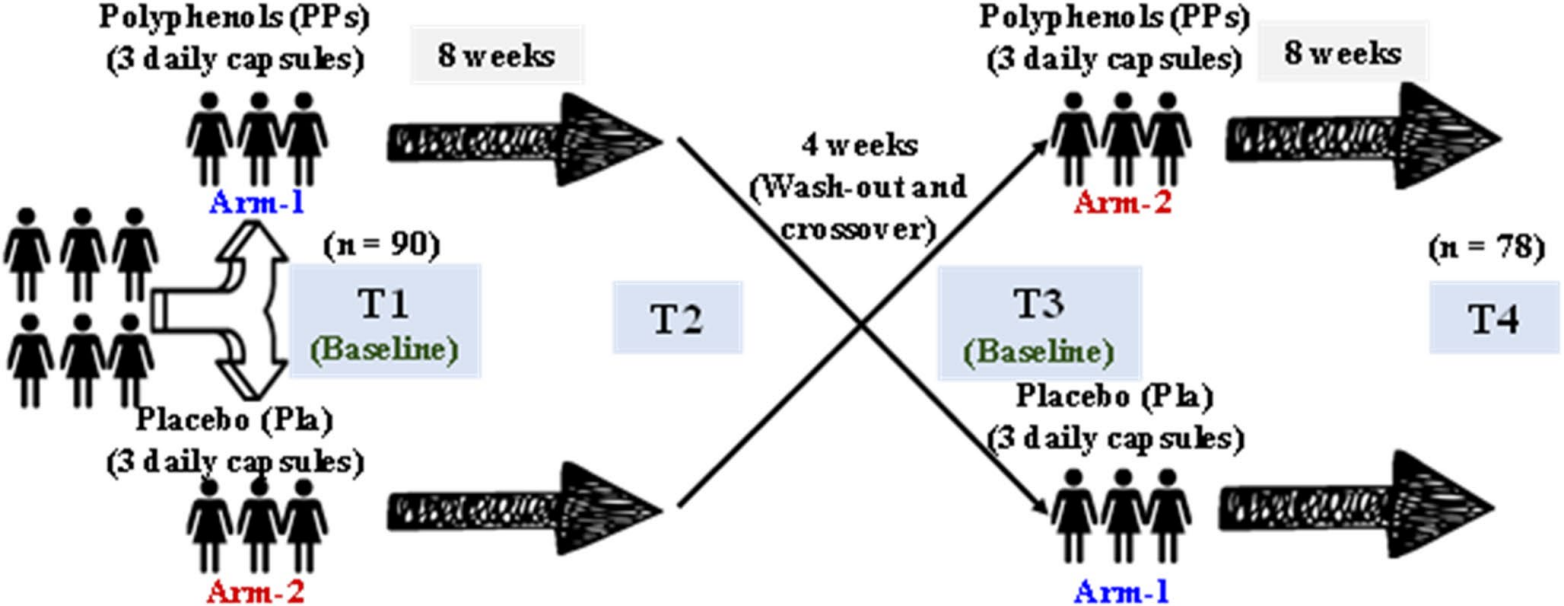
Schematic representation of the intervention study. Participants were randomised to receive either polyphenols (PPs) or the placebo (Pla) for 8 weeks, followed by a 4-week washout period, after which they crossed over to the alternate intervention for an additional 8 weeks. T1, T2, T3, and T4 denote study visits at which participants completed questionnaires (PREDIMED, IPAQ, protocol compliance, adverse events), provided urine and serum samples, and underwent anthropometric measurements. Daily intake consisted of three capsules of a polyphenol-rich extract mixture (PPs), delivering 150 mg *P. cuspidatum* extract (133.2 ± 10.1 mg *trans*-resveratrol), 1,200 mg pomegranate extract (312.0 ± 30.9 mg ellagitannins and ellagic acid), and 750 mg red clover extract (166.3 ± 27.3 mg isoflavones). The placebo consisted of three capsules containing 2100 mg microcrystalline cellulose

Postmenopausal women were randomly assigned in a 1:1 ratio to the treatment sequences using computer-generated numbers, without blocking or stratification. Allocation concealment was ensured by using indistinguishable capsules and a computer-generated randomisation list held by an independent researcher who had no access to the random-allocation sequence. The protocol was double-masked, and neither the participants nor the investigators responsible for follow-up and analysis were aware of the product allocation. Participants received either polyphenol-rich plant mixture (PPs) or the placebo (Pla) for 8 weeks, followed by a 4-week washout period, after which they crossed over to the alternate intervention for an additional 8 weeks (Fig. 1). Participants consumed the three capsules daily in the evening, as previously described [20, 21].

Throughout the metabotyping phase, intervention periods, and washout, participants followed a low‑polyphenol diet, supervised by a Nutritionist, as described previously [20, 21]. Dietary adherence was monitored using 3‑day dietary records, which were reviewed at each visit. Volunteers were instructed to avoid taking food supplements containing isoflavones, resveratrol, or pomegranate. Adherence to these restrictions was confirmed at each visit. The PREDIMED questionnaire evaluated the adherence to the Mediterranean diet.

Fasting blood and urine samples were collected at each study visit, and adverse events were recorded (Fig. 1). Harms were defined a priori as any adverse event related to the intervention, and were assessed systematically at each study visit using standardised questionnaires [20, 21].

No participants or members of the public were involved in the design, conduct, reporting, or dissemination plans of this research. No changes to the protocol, outcomes, or statistical analysis plan were made after trial commencement.

### Sample size

The required sample size for this two-period, two-sequence crossover study was calculated according to standard recommendations for crossover designs in nutrition research, with methodological considerations for continuous outcomes in clinical nutrition. Notably, a parallel design generally requires approximately 5 times as many participants as a crossover study to achieve equivalent statistical power and α [22]. An a priori calculation was conducted for the 2 × 2 crossover, with oxLDL as the primary outcome. Detecting a 25% change with 80% power and α = 0.05 required at least 12 participants, based on the expected within‑subject SD [22].

The recruitment target was increased to *n* = 90 to allow metabotype‑stratified analyses and account for unequal metabotype prevalence and an anticipated 15% dropout [20]. Metabotype distribution has already been characterised in this cohort before and after the intervention, as previously reported [20, 21].

### Treatment characterisation

The PPs intervention, consisting of *Polygonum cuspidatum*, pomegranate, and red clover extracts, was formulated as previously reported [20]. Each capsule (700 mg) contained standardised amounts of *trans*-resveratrol (50 mg, 98% purity), a punicalagin-enriched pomegranate extract (320 mg, 20% punicalagin), an ellagic acid (EA)-rich pomegranate extract (80 mg, 40% EA), and red clover extract (250 mg, 20% isoflavones). Placebo capsules contained 700 mg of microcrystalline cellulose and were visually identical to the PPs capsules [20]. Based on HPLC-ESI-IT-MS/MS (High‑Performance Liquid Chromatography - Electrospray Ionization - Ion Trap - Tandem Mass Spectrometry) analyses, the daily intake of three PPs capsules delivered 133.2 ± 10.1 mg of resveratrol, 312.0 ± 30.9 mg of ellagitannins (including punicalin and α- and β-punicalagins, along with free ellagic acid), and 166.3 ± 27.3 mg of isoflavones, comprising daidzein, genistein, formononetin, and biochanin A [20] (Fig. 1; Table S1).

### Blood sampling and biochemical analyses

Fasting peripheral blood samples were collected between 8 and 9 AM to minimise circadian variation. Samples were obtained at the 4 study visits (T1-T4, Fig. 1), and corresponding serum or plasma aliquots were stored at -80 °C until analysis. Biochemical variables were measured using an automated biochemical analyser (Cobas^®^ c703, Roche Diagnostics Int. Ltd, Rotkreuz, Switzerland) and included glucose, creatinine, albumin, bilirubin, Tchol, HDLc, LDLc, TGs, alanine aminotransferase (ALT), alkaline phosphatase (ALP), aspartate aminotransferase (AST), vitamin D, calcium, urea, total proteins, and urate. Thyroid-stimulating hormone (TSH), free T3 and T4 were determined using a Cobas^®^ e801 analytical unit (Roche Diagnostics). Coagulation variables (fibrinogen, prothrombin time, and activated partial thromboplastin time) were measured in citrated plasma with an ACL TOP 700 analyser (Instrumentation Laboratory, Lexington, MA, USA).

Bone markers (osteocalcin and β-C-terminal telopeptide of type I collagen (B-CTx)) were quantified by electrochemiluminescence immunoassay, and bone-specific ALP (BALP) by chemiluminescence immunoassay (LIAISON^®^ XL, DiaSorin S.p.A., Saluggia, Italy) at the hospital facilities. Haematological variables were determined using an automated haematology analyser (LH 780, Beckman Coulter, Fullerton, CA, USA).

### Identification and quantification of phenolic-derived metabolites, and metabotyping

Urinary phenolic-derived metabolites were analysed by ultra-high-performance liquid chromatography coupled with electrospray ionisation and quadrupole time-of-flight mass spectrometry (UPLC-ESI-QTOF-MS) as previously published [20]. Analytical validation, LOD/LOQ values, and creatinine normalisation procedures followed those previously described [20, 21].

Metabolite identification was based on direct comparison with available analytical standards whenever possible. Additional confirmation relied on spectral features, including molecular mass and fragmentation patterns. Quantification was performed using extracted ion chromatograms (EICs) employing validated UPLC-ESI-QTOF-MS methods under consistent analytical conditions [20, 23].

Metabotyping was determined from urinary sample analysis, with production groups defined by the summed levels of specific metabolites. Following ellagitannin intake, individuals can be classified into three metabotypes: UMA, producing only Uro-A; UMB, producing Uro-A, IsoUro-A and Uro-B; and UM0, characterised by undetectable urolithin production. Equol producers (EP) are individuals who produce equol from isoflavones, whereas equol non-producers (ENP) accumulate dihydrodaidzein. Lunularin producers (LP) can produce lunularin and, to a lesser extent, 4HDB from resveratrol. In contrast, lunularin non-producers (LNP) accumulate dihydroresveratrol [17]. MCs were defined a priori as all possible combinations of the three binary metabotypes (UMA/UMB/UM0, EP/ENP, LP/LNP), following the approach described in our previous studies [20, 21].

### ApoB, oxLDL, and LBP determination

Serum apolipoprotein B (Human ApoB ELISA Kit, ref. EH34RB), oxidised low-density lipoprotein (Human oxLDL ELISA Kit, ref. EEL074), and lipopolysaccharide-binding protein (Human LBP ELISA Kit, ref. EH297RB) were quantified using commercially available human ELISA kits from Invitrogen™ (Thermo Fisher Scientific, Waltham, MA, USA), according to the manufacturers’ specifications.

### Myeloperoxidase (MPO) activity

Serum myeloperoxidase (MPO) activity was measured using the human MPO Activity Assay Kit (ref. EEA016) from Invitrogen (Thermo Fisher Scientific), following the manufacturer’s instructions. The assay provides a functional evaluation of MPO by quantitatively determining its enzymatic activity.

### Statistical analyses

The effects of PPs were evaluated using a two-way repeated-measures ANOVA (2-w RM ANOVA) to assess treatment, period, and time effects (SigmaPlot v16.0, Systat Software, San Jose, CA, USA; jamovi v2.6.26, The jamovi project 2025, https://www.jamovi.org, Sydney, Australia). Data normality was verified with the Shapiro-Wilk test, and significant overall differences identified by the 2-w RM ANOVA were further examined using Bonferroni post hoc comparisons. When normality or sphericity assumptions were not met, the 2-w RM ANOVA was computed using Greenhouse-Geisser-adjusted degrees of freedom. All analyses were conducted using complete-case data. No imputation methods were applied. All efficacy analyses were performed in the per-protocol population, including only participants who completed both intervention periods (*n* = 78). Analyses were conducted both for the entire cohort as a single population and after stratification by individual metabotypes and metabotype clusters (MCs). Changes in blood lipids, oxLDL, ApoB, LBP, and the ratios (LDLc/ApoB, oxLDL/LDLc, oxLDL/ApoB) were estimated as least-squares means derived from the 2-w RM ANOVA model, which accounted for intra-subject variability inherent to the crossover design. Baseline values at the start of each intervention period (T1 and T3; Fig. 1) were compared between arms to assess baseline balance and check for potential carryover between treatment sequences [20, 21] (comparisons were performed using independent‑samples t‑tests or Mann-Whitney tests, depending on data distribution). Post-treatment changes (vs. baseline) in the above targets were correlated with BMI, chronological age, age at menopause onset, years since menopause, adherence to the Mediterranean diet (PREDIMED questionnaire), and physical activity levels (IPAQ scores) using Pearson/Spearman correlations and exploratory multiple linear regression models to evaluate possible covariate influence. Within-arm comparisons were performed using parametric (t-test) or non-parametric (Wilcoxon signed-rank) tests, depending on distribution. Effect sizes for within‑subject treatment differences were quantified using Cohen’s dz, calculated from the individual difference scores (ΔPPs - Δplacebo), and reported with their 95% confidence intervals [20].

Graphs were generated using GraphPad Prism 10.4.1 (GraphPad Software, San Diego, CA, USA) and SigmaPlot v16.0. Statistical significance was defined as *p < 0.05, **p < 0.01, and ***p < 0.001, with marginal significance considered for 0.05 < *p* < 0.1. The full study protocol and statistical analysis plan are available upon reasonable request from the corresponding author.

## Results

### Baseline characteristics of participants

A total of 185 women were contacted via the hospital. After the interviews, 90 women met the eligibility criteria and were enrolled. Table 1 shows the baseline characteristics of the completers (*n* = 78). Baseline characteristics of all recruited participants (*n* = 90) are provided in Table S2.

The distribution of polyphenol‑related metabotypes is shown in Table S3. UMA was the most frequent (71.9%), followed by EP and LP (50.6% each) and ENP and LNP (49.4%). Among clusters, MC7 (UMA + EP+LNP) represented 22.5% of participants, while MC2 (15.7%; UMA + ENP+LP), MC3 (15.7%; UMA + EP+LP), and MC5 (18%; UMA + ENP+LNP) were also prevalent. Clusters MC9–MC12, all containing UM0, were not detected (Table S3).

**Table 1:** Characteristics of completers (*n* = 78) at inclusion^a^

Baseline variable	Values
Age (years)	53.2 ± 3.1, (45−59)
BMI (kg/m^2^) Normal weight (%) Overweight (%) Obese (%)	26.2 ± 4.3, (20.4−37.4) 51.3 31.6 17.1
Weight (kg)	68.2 ± 11.0, (50.7−98.2)
Waist circumference (cm)	83.6 ± 10.1, (67.0−112.0)
Alcohol consumption (%) (Never / low)^b^	25.0 / 75.0
Age of menopause onset (years)	49.7 ± 3.6, (41−55)
Postmenopausal duration (years)	3.5 ± 2.8, (0.3−16)
Total cholesterol (mg/dL)	218.3 ± 28.0, (152−279)
LDL-cholesterol (mg/dL)	130.1 ± 27.4, (90−195)
HDL-cholesterol (mg/dL)	72.4 ± 21.7, (46−197)
Triglycerides (mg/dL)	81.0 ± 30.5, (36−184)
Glucose (mg/dL)	89.0 ± 11.2, (65−125)
ApoB (mg/dL)	98.0 ± 30.2, (44.9−151.1)
LDLc/ApoB	1.4 ± 0.6, (0.5−3.3)
oxLDL (mg/dL)	3.1 ± 1.5 10^−5^, (0.6−8.1) 10^−5^
oxLDL/LDLc^c^	0.3 ± 0.1, (0.1−1.0)
oxLDL/ApoB^c^	0.4 ± 0.2, (0.1−0.8)
LBP (µg/mL)	20.1 ± 7.8, (6−48)
ALP (U/L)	77.7 ± 19.4, (43–141)
ALT (U/L)	20.2 ± 8.3, (5–54)
AST (U/L)	21.3 ± 4.7, (14–37)
LDH (U/L)	182.7 ± 24.8, (135–258)
TSH (µU/mL)	1.9 ± 1.2, (0.04–5.7)
Free T4 (ng/dL)	1.2 ± 0.3, (0.7–1.7)
Free T3 (pg/mL)	3.2 ± 0.4, (2.5–3.9)
Fibrinogen (g/L)	442.3 ± 62.7, (324–615)
Prothrombin time (s)	10.8 ± 0.6, (9.8–12.3)
International normalised ratio (INR)	0.98 ± 0.05, (0.9–1.1)
Activated partial thromboplastin time (s)	31.4 ± 2.5, (27.6–40.8)
Calcium (µg/L)	9.6 ± 0.4, (8.7–10.5)
B-CTX (µg/L)	0.6 ± 0.2, (0.1–1.0)
BALP (µg/L)	15.2 ± 5.0, (7.9–32.0)
Vitamin D (µg/L)	31.0 ± 12.1, (15.2–86.1)
Creatinine (mg/dL)	0.72 ± 0.1, (0.5–1.0)
Bilirubin (mg/dL)	0.5 ± 0.3, (0.2–1.4)
Urate (mg/dL)	4.2 ± 0.8, (2.7–7.0)
Total proteins (g/dL)	7.2 ± 0.4, (6.4–9.8)
Albumin (g/dL)	4.6 ± 0.2, (4.2–4.9)
Urea (mg/dL)	34.5 ± 7.6, (18–51)
Adherence to Mediterranean diet (PREDIMED) Low, medium, high (%)	16.7 / 73.1 / 10.2
IPAQ score Low, medium, high (%)	59.1 / 33.7 / 7.2

^a^Values are shown as mean ± SD and range, or percentage. ^b^Low refers to ≤ 10 g of alcohol consumption per day [24]. ^c^oxLDLc/LDLc and oxLDL/ApoB ratios (dimensionless) have been multiplied by 10^6^ for readability

PREDIMED adherence categories were calculated from the 14-item Mediterranean diet questionnaire (score range 0–14) and classified as low (≤ 5), medium (6–9), and high (≥ 10) [25]. IPAQ categories were derived from the International Physical Activity Questionnaire-Short Form [26], and were normalised to a 0−100 scale for readability and classified as low (0−33), moderate (34−66), and high (67−100) physical activity levels

At inclusion, the 78 postmenopausal women that completed the intervention had a mean age of 53 years, with menopause onset at 50 and a postmenopausal duration of ∼4 years (Table 1). The mean BMI was 26 kg/m², with 50% classified as normal weight, 36% overweight, and 14% obese. Most participants reported low alcohol consumption, moderate adherence to the Mediterranean diet, and predominantly low physical activity. The lipid profile indicated mild hypercholesterolaemia (Tchol averaging 218 mg/dL, LDLc 130 mg/dL, and ApoB 98 mg/dL), with high HDLc (72 mg/dL), and normal TGs (81 mg/dL). Glucose, liver and thyroid function, renal markers, and bone turnover markers were within normal ranges. Overall, the cohort showed borderline dyslipidemia but otherwise preserved metabolic, hepatic, renal, and bone health.

### Polyphenol supplementation increases blood lipids, with age-related increases

**Fig. 2:**
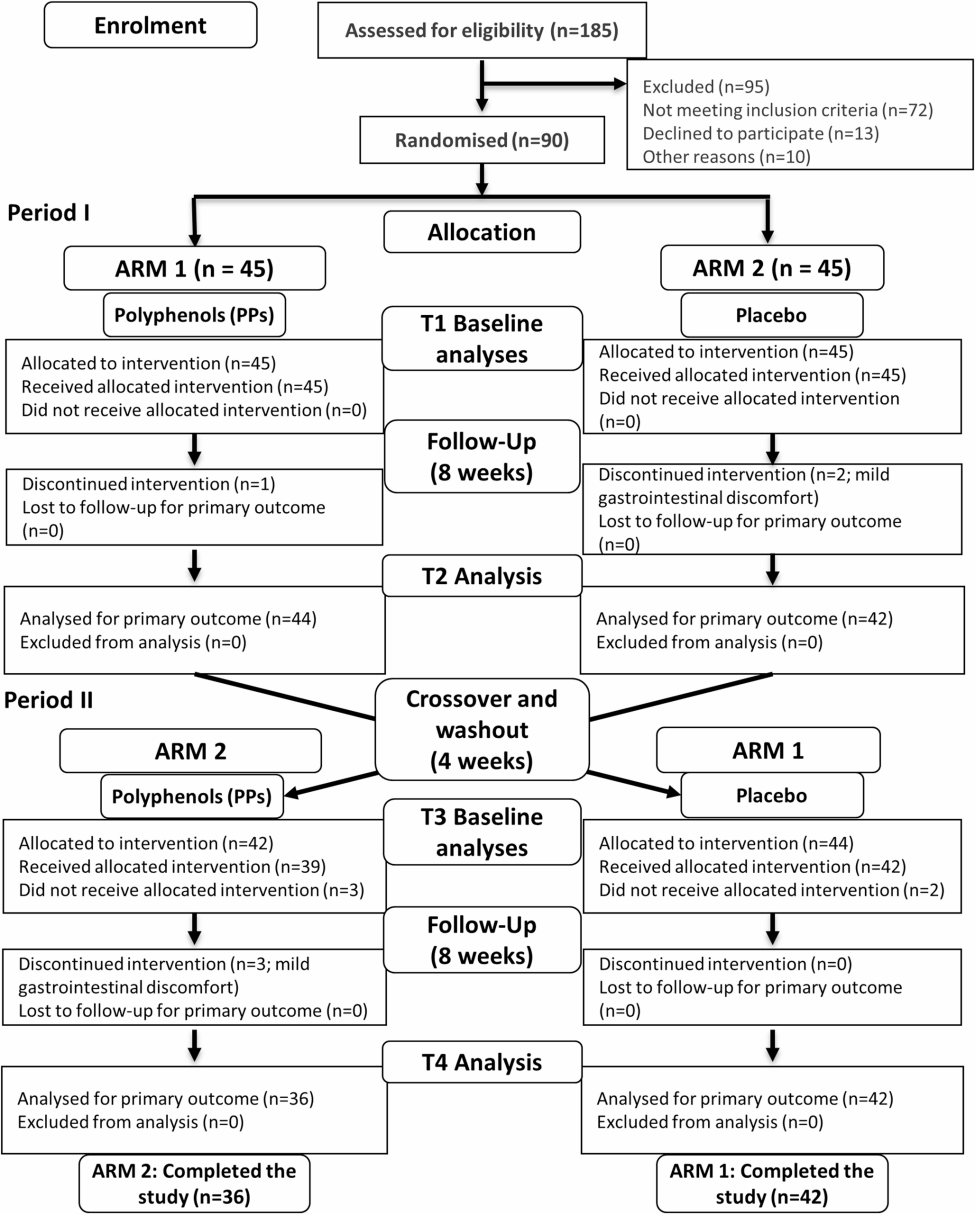
CONSORT diagram. Flow chart illustrating participant enrollment, recruitment, randomisation, treatment assignments, and crossover, with subsequent analysis across the study period

Seventy-eight participants completed the 20-week intervention. The CONSORT diagram of the trial is shown in Fig. 2. Adherence to the diet and capsule protocol was 100%, with minimal deviations. Three participants reported mild gastrointestinal discomfort during the polyphenol phase, and two during the placebo phase (Fig. 2). No serious adverse events occurred.

After 8 weeks of polyphenol supplementation (PPs), slight changes in metabotype distribution were detected (Table S3). UMB increased slightly, with small shifts in UMA, EP, and LP. MC frequencies shifted accordingly. MC3 expanded to 20.5% and overtook MC7 (19.2%), which had been the most common at baseline. In addition, clusters MC1 and MC4 showed growth (12.8% and 14.1%, respectively), whereas MC2 decreased to 9.0% (Table S3).

**Fig. 3:**
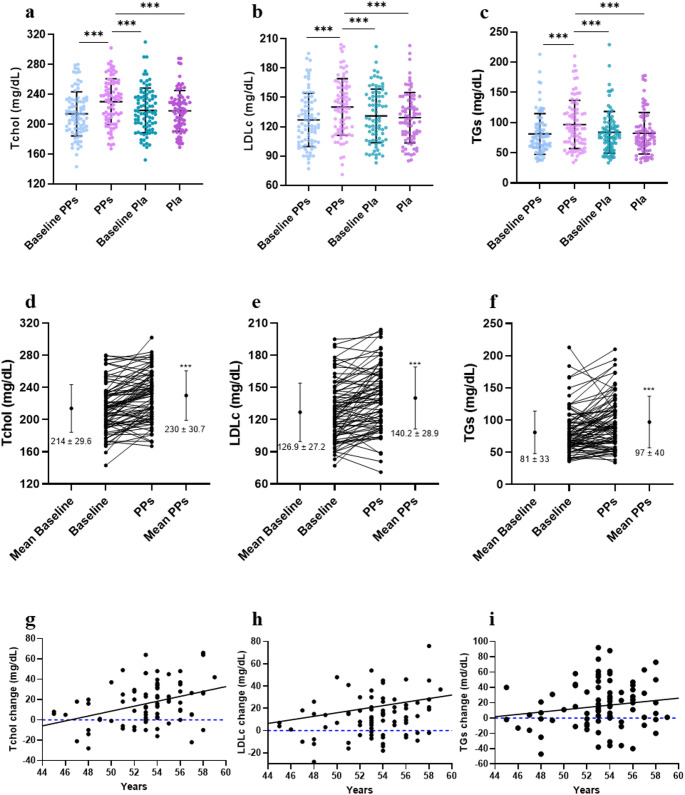
Changes in Tchol **a**, LDLc **b**, and TGs **c** after polyphenol (PPs) or placebo (Pla) consumption; ***p < 0.001. Panels **d**, **e**, and **f** show mean individual participant changes in Tchol, LDLc, and TGs, respectively, following PPs intake compared with baseline (***p < 0.001). Changes in Tchol **g** (*r* = 0.4, *p* < 0.001), LDLc **h** (*r* = 0.4, *p* < 0.001), and TGs **i** (*r* = 0.16, *p* = 0.14) as a function of age after PPs supplementation in postmenopausal women (some points are superimposed due to overlapping values). The blue dashed line indicates no change; values above it represent an increase, and values below it represent a decrease

Following the intervention, no significant changes were observed in LBP, glucose, thyroid hormones, transaminases, bone turnover markers, coagulation, or haematological variables (data not shown), and were independent of metabotypes. Likewise, no changes were observed in BMI, weight, and waist circumference. In contrast, a significant mean increase (*p* < 0.001) in Tchol (7%), LDLc (9.5%), and TGs (16%) was observed after PPs supplementation, affecting ~ 80% of completers compared with baseline and placebo, with no evidence of carryover effect (Fig. 3). Baseline values at the start of the second period (T3) returned to levels comparable to those observed before the first intervention (T1), indicating that the effects observed during the first period did not persist into the subsequent period.

Effect sizes were moderate to large (Tchol: Cohen’s dz = 0.73, 95% CI 0.48–0.98; LDLc: 0.61, 95% CI 0.37–0.85; TGs: 0.63, 95% CI 0.39–0.87). This lipid elevation was observed at both recruitment sites, excluding analytical errors (Figs. S1a, b). It was also independent of baseline values, as shown by strong correlations between pre- and post-intervention levels (Tchol *r* = 0.82; LDLc *r* = 0.78; TGs *r* = 0.62; all *p* < 0.001), indicating a consistent increase across the cohort rather than a disproportionate rise among women with lower initial blood lipids (Fig. S1c–e).

Importantly, increases in Tchol and LDLc were positively correlated with age (*p* < 0.001), whereas the increase in TGs did not reach statistical significance (*p* = 0.14). In the ~ 20% of participants who showed slight reductions in Tchol and LDLc, these decreases were independent of advancing age (Fig. 3g–i). Blood lipid changes were not linked to metabotypes or their MCs (Table S4 Moreover, no correlations were found with BMI, age at menopause, years since menopause, adherence to the Mediterranean diet, or physical activity (Table S5).

### Polyphenol supplementation reduces oxLDL and improves LDLc/ApoB and oxLDL-related ratios

**Fig. 4:**
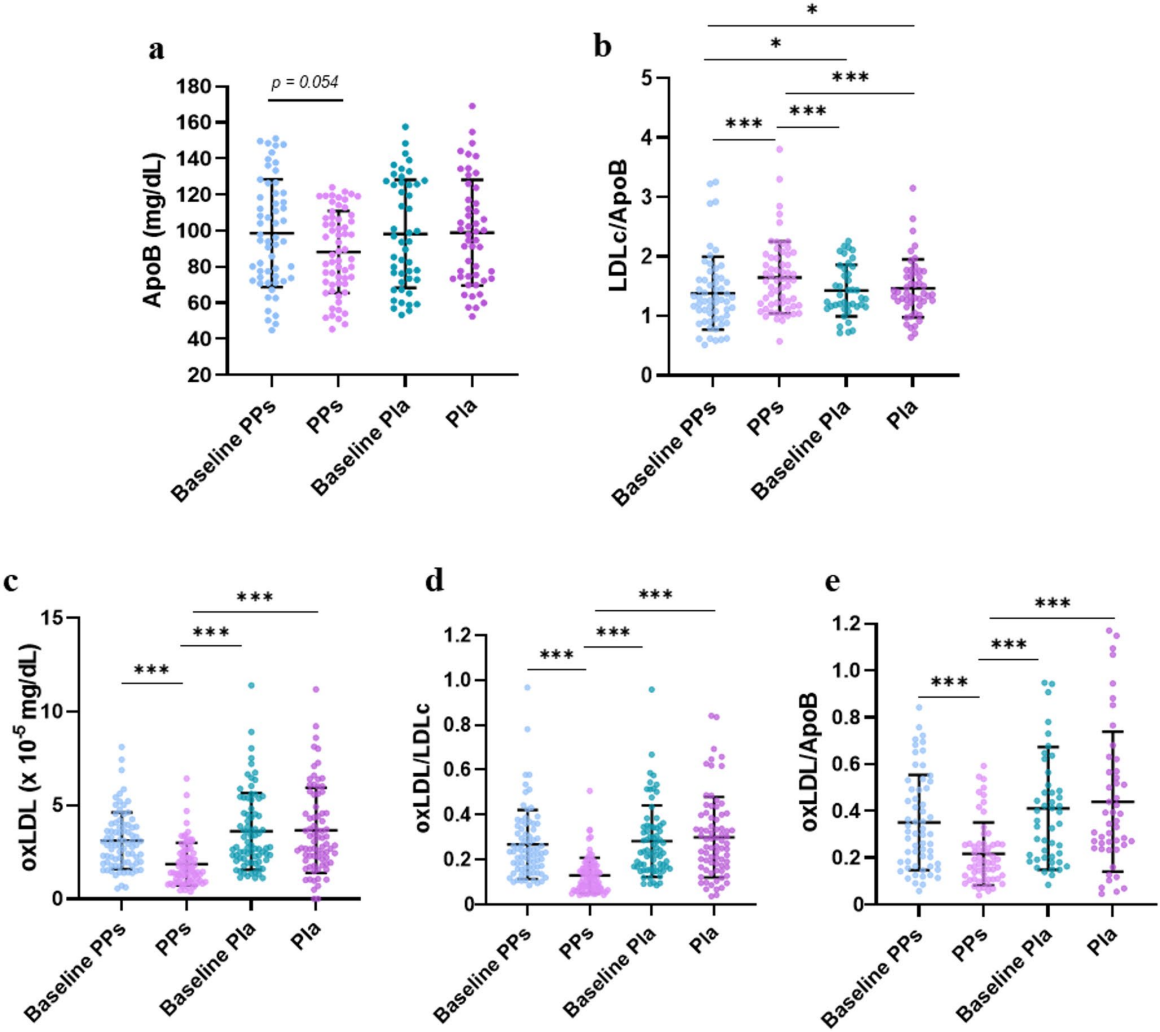
Changes in ApoB **a**, LDLc/ApoB **b**, oxLDL **c**, oxLDL/LDLc **d**, and oxLDL/ApoB **e** after polyphenol (PPs) or placebo (Pla) consumption in the full cohort (***p < 0.001; *p < 0.05)

Next, we aimed to assess the atherogenic quality of LDL particles to determine whether the increase in Tchol concentration was accompanied by a rise in particle number. ApoB showed a marginal reduction (*p* = 0.054) after PPs intake. This borderline reduction corresponded to an ≈ 11% net decrease in ApoB and a moderate within‑subject effect (Cohen’s dz = − 0.54; 95% CI − 0.77 to − 0.30), indicating that the rise in LDLc concentration was not accompanied by an increase in LDL particle number. This pattern is consistent with a shift toward larger, less atherogenic LDL particles (Fig. 4a). This was supported by a significant increase in the LDLc/ApoB ratio (*p* < 0.001), a proxy for cholesterol load per LDL particle, which increased significantly following PPs consumption (*p* < 0.001, Cohen’s dz = 0.58; CI 95% [0.34, 0.82]) (Fig. 4b).

Circulating oxLDL decreased by 39% (*p* < 0.001; Cohen’s dz = − 0.71, 95% CI − 0.96 to − 0.46) (Fig. 4c). The oxLDL/LDLc ratio also declined (*p* < 0.001; Cohen’s dz = − 1.70, 95% CI − 2.04 to − 1.36) (Fig. 4d), indicating a lower proportion of oxLDL relative to total LDLc, and the oxLDL/ApoB ratio showed an even larger reduction (*p* < 0.001; Cohen’s dz = − 3.97, 95% CI − 4.63 to − 3.31) (Fig. 4e), indicating a markedly lower oxidative burden per LDL particle and a less atherogenic LDL profile.

Finally, no significant associations were found between metabolite amounts, metabotypes or MCs and changes in oxLDL or the related ratios (Table S6). These null associations for oxLDL were consistent with our previous observations for Tchol, LDLc and TGs, which also showed no relationship with metabotypes or MCs. However, unlike oxLDL, these lipid variables were positively correlated with age.

### Metabotyping reveals that LDL oxidative status improves more in equol producers and in the MC7 cluster

Previously, we observed that increases in Tchol, LDLc, and TGs were predominant among most participants and were positively correlated with age, but not with metabotype or MCs. We then sought to determine whether the improvement in LDLc oxidative quality after PPs intake followed a similar pattern or could be metabotype-dependent. OxLDL concentrations did not correlate with BMI, age, age at menopause, or years since menopause (data not shown).

After stratifying by metabotypes, oxLDL decreased across all groups, but to different extents. These differences reflect within‑metabotype responses (fold‑changes and within‑group *p*‑values) and were not derived from direct statistical comparisons between metabotypes, as the study was not powered for between‑metabotype contrasts. Reductions were evident in UMA (1.8-fold, *p* < 0.001) and UMB (1.7-fold, *p* < 0.001), and similarly in LP (1.7-fold, *p* < 0.001) and LNP (1.9-fold, *p* < 0.001). In equol non-producers (ENP), the decrease was smaller (1.2 fold) and non-significant.

**Fig. 5:**
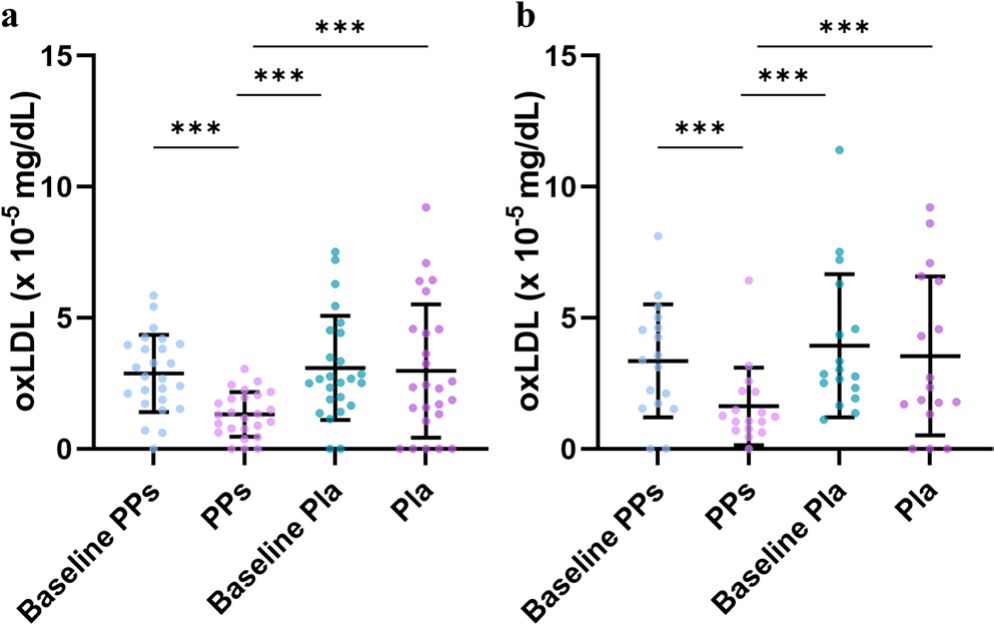
Metabotype-dependent changes in oxLDL after consuming polyphenols (PPs) or the placebo (Pla). **a** EP, equol producers, and **b** MC7, UMA + EP+LNP (***p < 0.001)

Consistently, MCs containing the ENP metabotype showed modest or non‑significant reductions. In contrast, equol producers (EP) and the MCs that included them, particularly MC7 (UMA + EP+LNP), showed the strongest responses, with reductions of 2.3‑fold in EP and 2.5‑fold in MC7 (*p* < 0.001; Fig. 5). Effect sizes were very large in both groups (EP: Cohen’s dz = − 4.49; 95% CI − 5.46 to − 3.52; MC7: Cohen’s dz = − 3.49; 95% CI − 4.83 to − 2.15), indicating a particularly pronounced response for these women. However, the MC7 result should be interpreted with caution, given its small sample size (*n* = 15).

### Effect of polyphenol intake on MPO activity

**Fig. 6:**
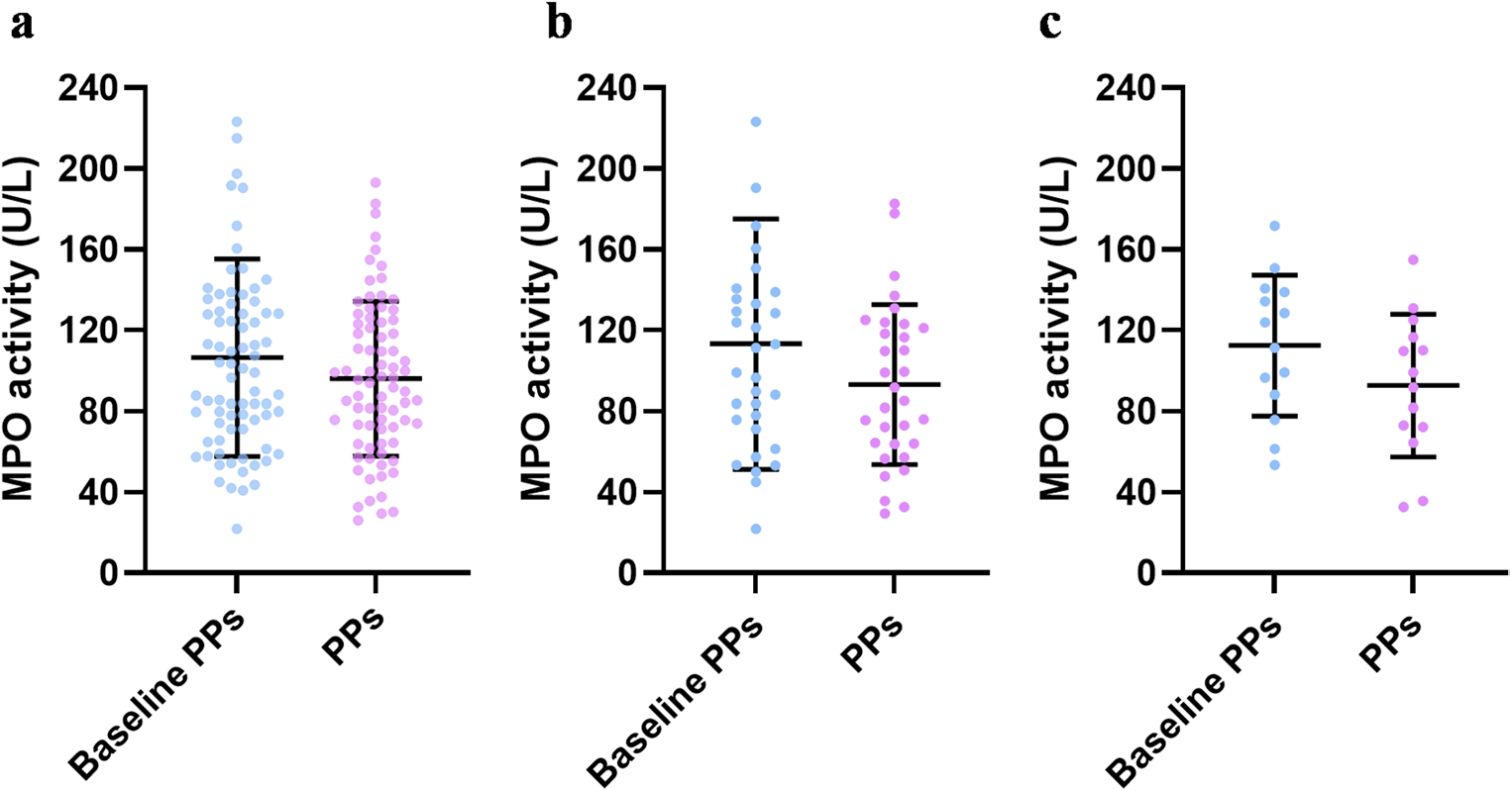
Change in MPO activity at baseline and after consuming PPs. **a** Full cohort (*n* = 78, *p* = 0.08), **b** LNP, lunularin non-producers (*p* = 0.06), and **c** MC7, UMA + EP+LNP (*p* = 0.24)

Serum MPO activity was assessed at baseline and after PPs intake as an exploratory mechanistic marker to contextualise the reductions observed in oxLDL. In the whole cohort, MPO showed a downward trend reaching marginal significance (*p* = 0.08) (Fig. 6a).

Stratification by metabotypes revealed similar tendencies in EP and UMA women, with a slight, non-significant decrease observed (Fig. S2), and LNP participants showing a marginally significant decrease (*p* = 0.06) (Fig. 6b). MC7 (UMA + EP+LNP) also showed a consistent reduction, although not statistically significant (*p* = 0.24) (Fig. 6c), likely due to the small sample size (*n* = 15). These exploratory findings suggest that MPO modulation may contribute to the metabotype-dependent reduction in oxLDL, although dedicated mechanistic studies are needed to confirm this.

## Discussion

We report here that high-dose polyphenol supplementation increased Tchol, LDLc, and TGs in non‑medicated postmenopausal women, with a consistent pattern across most participants. Nevertheless, despite this rise, LDL oxidative status improved. The precise mechanisms underlying this dissociation were not addressed and warrant further investigation. Analyses of ApoB and related ratios (LDLc/ApoB, oxLDLc/LDLc, oxLDL/ApoB) indicated fewer, larger, and less oxidised LDL particles. These findings align with the concept that cardiovascular risk is influenced by both lipid concentrations and the quality and oxidative state of lipoprotein particles [27, 28]. This discordance suggests that each LDL particle carried more cholesterol but was less atherogenic due to lower oxidation. The LDLc/ApoB ratio, a proxy for cholesterol content per particle, has prognostic relevance and supports evaluating LDL particle quality in addition to concentration [27−29]. Current dyslipidaemia guidelines emphasise that atherogenic risk is determined primarily by the number of ApoB‑containing particles rather than by LDLc concentration alone [30].

MPO, evaluated as an exploratory marker, showed a marginal overall reduction (0.05 < *p* < 0.1). This modest decrease is consistent with a potential involvement of MPO-related processes in LDL oxidation, although other pathways are likely involved.

Age and dosage appear to modulate these effects on blood lipids. Previous studies found stronger lipid responses in women under 65 or with higher doses over longer interventions, consistent with our cohort [10]. Isoflavones and other polyphenols, including resveratrol, have sometimes produced neutral or adverse changes in HDLc, LDLc, and Tchol, highlighting concerns about dosage, duration, and individual variability [8, 11, 14–16]. 

Mechanistically, the paradox of increased LDLc with improved LDL quality may reflect polyphenol-induced lipoprotein remodelling. By modulating cholesteryl ester transfer protein, lipases and LDL receptor activity, polyphenols and their microbial metabolites can alter particle size and oxidative susceptibility [9, 27, 29−32]. In estrogen-deficient conditions, this may redistribute cholesterol without increasing particle number or atherogenic burden. Thus, preferential LDL receptor handling of less oxidised LDL could permit higher circulating LDLc without increased risk [33, 34], while isoflavones, acting as selective estrogen receptor modulators, may contribute to heterogeneous lipid responses [35].

The decline in estrogen after menopause creates a systemic pro-oxidant environment that impairs endothelial nitric oxide production, mitochondrial efficiency, and antioxidant enzyme expression [36]. In this context, LDL particles become more vulnerable to oxidation, and oxLDL strongly correlates with endothelial dysfunction and atherosclerosis [37]. MPO is a key enzymatic contributor to LDL oxidation in vivo [38]. Polyphenols appear to counterbalance these changes by scavenging free radicals and modulating redox-sensitive signalling, mitochondrial activity, and inflammatory enzymes involved in LDL oxidation [39]. Accordingly, the tendency toward lower MPO activity in specific metabotypes suggests partial modulation of oxidative pathways by polyphenol intake [27, 38, 40].

Beyond oxidative stress, estrogen deficiency also reshapes hepatic lipid handling. Lower LDL receptor activity, dysregulated lipase function, and enhanced VLDL secretion prolong lipoprotein circulation [41]. Polyphenols may interact with these pathways, activating hepatic cholesterol-sensing mechanisms and redistributing lipoprotein particles without proportionally enhancing receptor-mediated clearance [42]. Under such conditions, the rise in Tchol, LDLc, and TGs likely reflects particle redistribution and prolonged residence time rather than increased atherogenic burden, particularly when particle number and oxidation are reduced [36, 40].

Taken together, these findings suggest that polyphenols in postmenopausal women may act less as classical lipid-lowering agents and more as modulators of LDL quality and oxidative status [27, 29, 43]. Thus, the increases in circulating lipids may reflect the combined effects of lipoprotein remodelling and the metabolic context of estrogen deficiency, and the concurrent changes in ApoB, LDLc/ApoB and oxLDL‑related ratios indicate that the atherogenic profile cannot be inferred from lipid concentrations alone.

In certain individuals, specific gut microbes or microbial consortia transform polyphenols into unique metabolites. This metabolic diversity underlies the concept of polyphenol-related gut microbiota metabotypes, which classify people by their capacity to generate compounds such as urolithins A or B, lunularin, or equol from ellagitannins, resveratrol, and isoflavones, respectively, each with potential distinct effects on health [17]. Accordingly, previous reports describe metabotype-dependent effects of polyphenol supplementation on blood lipid modulation [18, 44, 45], intestinal permeability [46], and quality of life and TMAO production in postmenopausal women [20, 21].

In this study, gut microbiota metabotypes strongly modulated reductions in LDL oxidation, with equol producers (EP) and the MC7 cluster showing the greatest decreases. This metabotype-dependent response helps explain the heterogeneity in previous trials and emphasises the importance of considering individual metabolic signatures when evaluating polyphenol effects. Interestingly, the increase in circulating lipids did not clearly correlate with urinary concentrations of individual phenolic metabolites, reflecting the complexity of polyphenol metabolism and the limitations of single-time-point measures following long-term interventions. Biological activity likely depends on network-level interactions rather than single metabolites acting in isolation [9, 17, 19, 47, 48].

A distinctive strength of this study is the recruitment of non-medicated postmenopausal women, avoiding confounding from drugs such as statins, antihypertensives, hormone therapy, or psychotropic medications. This allowed the effects of polyphenols to be observed with high internal validity. The placebo-controlled crossover design and excellent adherence reduced inter-individual variability and enabled the detection of clear lipid and oxidative responses. Moreover, both the increase in circulating lipids and the reduction in oxLDL reverted after a 4-week washout, supporting a direct causal relationship with supplementation and reflecting dynamic metabolic adaptation rather than long-term or irreversible changes. This reversibility underscores the plasticity of lipid metabolism and the rapid responsiveness of redox-sensitive biomarkers to dietary interventions [49]. Although the intervention period was relatively short (8 weeks), primary endpoints such as oxLDL, ApoB, and LDLc/ApoB responded rapidly and reverted after washout, indicating that the duration was sufficient to capture the intended effects. The minimum time required to implement these changes has not been established, but the responses observed here indicate that 8 weeks were sufficient. Finally, no serious adverse events were reported, supporting the intervention’s overall safety.

We must acknowledge several limitations. The unexpected age‑dependent increase in Tchol, LDLc, and TGs after PPs supplementation vs. placebo warrants further investigation, as the study was not designed to elucidate mechanisms underlying this response. Other age‑related physiological factors not assessed in this trial may also contribute to this pattern, and this should be explored in future studies. The oxidative stress assessment was limited to oxLDL and MPO, thereby restricting mechanistic interpretation and precluding full characterisation of the oxidative pathways involved in LDL modification, which cannot be comprehensively addressed in a single study. Some metabotype‑stratified analyses were constrained by sample size, particularly in less prevalent metabotypes and clusters. These constraints affected only exploratory comparisons and did not compromise the primary outcome or the predefined metabotype analyses. In addition, the absence of UM0 individuals in this cohort reflects the limited sensitivity of the classical 3‑day ellagitannin metabotyping protocol, which may fail to detect low levels of urolithin production. For this reason, the absence of UM0 in this cohort does not restrict the interpretation or generalisation of the present findings. Lipoprotein remodelling pathways (CETP, LPL, hepatic lipase, LDL receptor activity) were not directly measured, so the interpretation that polyphenol supplementation promotes larger, less oxidised LDL particles relies on circulating biomarkers rather than on functional assays. Furthermore, the gut microbiota composition and functionality underlying each metabotype or cluster were not characterised, and related analyses are in progress, limiting the ability to link specific microbial communities to metabolites that could influence lipoprotein-modifying pathways.

The intervention combined ellagitannins, isoflavones, and resveratrol to enable metabotype-based clustering, thereby preventing the separate assessment of each polyphenol class’s contribution. Although the dose was relatively high, it remains feasible for nutraceutical use. Thus, dose-response studies are needed to establish whether similar improvements in LDL quality can be achieved with lower intakes. Finally, urinary phenolic metabolites were measured at a single timepoint per intervention period, which may not fully capture temporal variability in microbial metabolism.

## Conclusion

High-dose polyphenol supplementation in non-medicated, mildly hyperlipidemic postmenopausal women increased circulating Tchol, LDLc, and TGs compared with placebo, particularly with advancing age, yet simultaneously improved LDL particle quality, as reflected by lower oxLDL and favourable ApoB- and oxLDL-related ratios. Thus, the combined intake of ellagitannins, isoflavones, and resveratrol can dissociate lipid concentration from lipoprotein atherogenicity. The reduction in LDL oxidation was strongly metabotype-dependent, with equol producers (EP) showing the greatest response and the MC7 cluster (UMA + EP+LNP) also exhibiting a marked decrease, partially matching the exploratory tendencies observed for MPO. Overall, integrating lipid, oxidative, and metabotype profiling provides a more nuanced understanding of inter-individual variability in polyphenol responses and supports metabotype-based precision approaches in postmenopausal women.

## Supplementary Information

Below is the link to the electronic supplementary material.

Supplementary Material 1

## Data Availability

The datasets generated and analysed during the current study cannot be made publicly available due to General Data Protection Regulation (GDPR) requirements and Ethics Committee restrictions, as full anonymisation of individual-level health data cannot be ensured. Deidentified data may be made available upon reasonable request to the corresponding sponsor (J.C.E.) and subject to institutional and ethics committee approval.
