# Supplementary material for: Polyphenols increase circulating lipids but improve LDL particle quality and reduce LDL oxidation in postmenopausal women: metabotype- and age-dependent effects in a randomised, placebo-controlled crossover trial
Source: Eur J Nutr. 2026 Jun 29;65(5):177. doi: 10.1007/s00394-026-04027-2 (PMC13315490; doi:10.1007/s00394-026-04027-2)
Supplement: Supplementary file 1 — Supplementary Material 1 [file 394_2026_4027_MOESM1_ESM.pdf]

SUPPLEMENTARY INFORMATION

Polyphenols increase circulating lipids but improve LDL particle quality and reduce LDL oxidation in postmenopausal women: Metabotype- and age-dependent effects in a randomised, placebo-controlled crossover trial

María García-Nicolás,[1#]* María Paula Jarrín-Orozco,[1#] María Romo-Vaquero,[1] Concepción Carrascosa,[2] Francisco Avilés-Plaza,[3] Miriam Martínez-Villanueva,[3] José Antonio Noguera,[3] María Ángeles Ávila-Gálvez,[1] and Juan Carlos Espín[1]*

1 Laboratory of Food & Health, Research Group on Quality, Safety, and Bioactivity of Plant Foods, CEBAS-CSIC, 30100 Campus de Espinardo, Murcia, Spain
2 Department of Obstetrics and Gynaecology, Virgen de La Arrixaca University Hospital, El Palmar, 30120, Murcia, Spain
3 Institute of Biomedical Research (IMIB-Arrixaca), Hospital Clínico Universitario Virgen de la Arrixaca, 30120 Murcia, Spain

*M. García-Nicolás: mgnicolas@cebas.csic.es; J.C. Espín: jcespin@cebas.csic.es

[#]These authors contributed equally

Supplementary Materials

The polyphenols and gut microbial standards, including *trans*-resveratrol, ellagic acid, daidzein, genistein, formononetin, biochanin A, equol, and hesperetin, were purchased from Sigma-Aldrich (St. Louis, MO, USA), whereas equol 7-*O*-glucuronide and *O*-demethylangolensin (ODMA) were acquired from LGC Standards (Barcelona, Spain). Resveratrol 4'-*O*-sulfate, resveratrol 3-*O*-glucuronide, dihydroresveratrol 3-*O*-glucuronide, resveratrol 3-*O*-sulfate, Urolithin A (Uro-A), B (Uro-B), Isourolithin A (IsoUro-A), Uro-A 3-*O*-glucuronide, Uro-A 8-*O*-glucuronide, Uro-B glucuronide, Uro-A sulfate, Uro-B sulfate, IsoUro-A 3-*O*-glucuronide, IsoUro-A 9-*O*-glucuronide, lunularin, 4-hydroxydibenzyl (4HDB), lunularin glucuronide, lunularin sulfate, 4HDB glucuronide and 4HDB sulfate were obtained as described by Jarrín-Orozco et al. 2026. All reagents and metabolites were of at least 97% purity. Ultrapure Millipore water was used throughout all experiments.

Table S1 Phenolic compounds in the plant extract mixture (PPs)

Extracts phenolics	RT	*m/z*⁻	MS/MS	λ_{max}	mg/g extract	mg/capsule
**Red clover**						
Daidzein	32.33	253	225/209	248/304	5.20±0.61	3.64±0.43
Genistein	37.15	269	241/225	260/324	4.86±0.15	3.41±0.10
Formononetin	40.80	267	252/235/211	250/304	43.81±1.60	30.67±1.12
Biochanin A	43.15	283	268/251/227	260/332	25.34±10.63	17.74±7.44
					**Σ Isoflavones**	**55.45±9.10**
P. cuspidatum						
trans-Resveratrol	30.84	227	185/157	306	63.42±4.80	44.39±3.36
**Pomegranate**						
Punicalin	12.76	781	721/601/299	258/380	11.47±0.92	8.03±0.65
Punicalagin α	15.08	1083	781/721/601	258/372	48.15±8.75	33.71±6.13
Punicalagin β	17.08	1083	781/721/601	258/372	48.20±6.24	32.16±2.28
Ellagic acid	25.68	301	257/229/185	254/360	42.81±1.81	29.97±1.27
					**Σ Ellagitannins and ellagic acid**	**103.9±10.32**
				**Total phenolics**	**203.70±22.80**	

The plant extract mixture (PPs) was characterised using High-Performance Liquid Chromatography coupled to Electrospray Ionisation Ion-Trap Tandem Mass Spectrometry (HPLC-ESI-IT-MS/MS), following the protocol described by Ávila-Gálvez et al. [23]. Compound identification relied on retention behaviour, UV absorption spectra, mass-to-charge ratios, MS/MS fragmentation profiles, and comparison with authentic standards when available. Direct standard matching allowed the identification of *trans*-resveratrol (quantified at 310 nm), ellagic acid (360 nm), punicalagin α and β (360 nm), daidzein (270 nm), and genistein (270 nm). Punicalin was determined as the combined signal of its α- and β-isomers at 360 nm. Formononetin and biochanin A (both at 270 nm) were quantified using daidzein as the reference compound. Results are reported as mean ± SD (n=5). RT denotes retention time

Table S2 Characteristics of postmenopausal women (n = 90) at inclusion[a]

Baseline variable	Values
Age (years)	53.2 ± 3.3, (45–59)
BMI (kg/m^2)	26.0 ± 4.3, (19.0–37.4)
Normal weight (%)	50.0
Overweight (%)	35.6
Obese (%)	14.4
Weight (kg)	67.4 ± 10.9, (47.7–98.9)
Waist circumference (cm)	83.1 ± 10.4, (63–112)
Alcohol consumption (%) (Never / low)[b]	32.3 / 67.7
Age of menopause onset (years)	49.8 ± 3.7, (40–56)
Postmenopausal duration (years)	3.9 ± 3.5, (0.3–14)
Total cholesterol (mg/dL)	214.0 ± 29.6, (143–280)
LDL-cholesterol (mg/dL)	126.9 ± 27.2, (77–195)
HDL-cholesterol (mg/dL)	68.2 ± 11.8, (46–98)
Triglycerides (mg/dL)	81.0 ± 33.0, (46–213)
Glucose (mg/dL)	90.4 ± 10.9, (69–125)
ApoB (mg/dL)	99.1 ± 30.7, (45–167)
LDLc/ApoB	1.4 ± 0.6, (0.5–3.2)
oxLDL (mg/dL)	3.2 ± 1.6 10^{-5}, (0.5–8.1) 10^{-5}
oxLDL/LDLc[c]	0.3 ± 0.1, (0.1–0.9)
oxLDL/ApoB[c]	0.4 ± 0.2, (0.1–0.8)
LBP (μg/mL)	20.3 ± 8.8, (6–49)
ALP (U/L)	77.6 ± 18.7, (43–141)
ALT (U/L)	20.1 ± 8.3, (5–54)
AST (U/L)	21.2 ± 5.1, (14–39)
LDH (U/L)	182.9 ± 23.9, (135–258)
TSH (μU/mL)	1.9 ± 1.1, (0.04–5.7)
Free T4 (ng/dL)	1.2 ± 0.2, (0.7–1.7)
Free T3 (pg/mL)	3.2 ± 0.4, (2.5–3.9)
Fibrinogen (g/L)	440.7 ± 61.5, (324–615)
Prothrombin time (s)	10.8 ± 0.6, (9.3–12.3)
International Normalised Ratio (INR)	0.97 ± 0.06, (0.8–1.1)
Activated partial thromboplastin time (s)	31.3 ± 2.5, (27.1–40.8)
Calcium (μg/L)	9.6 ± 0.4, (8.7–10.5)
B-CTX (μg/L)	0.6 ± 0.2, (0.1–1.0)
Osteocalcin (μg/L)	28.6 ± 8.7, (8.3–55.3)
BALP (μg/L)	15.1 ± 4.8, (7.9–32.0)
Vitamin D (μg/L)	31.6 ± 11.9, (15.2–86.1)
Creatinine (mg/dL)	0.7 ± 0.1, (0.5–0.9)
Bilirubin (mg/dL)	0.5 ± 0.2, (0.1–1.4)
Urate (mg/dL)	4.2 ± 0.8, (2.7–7.0)
Total proteins (g/dL)	7.2 ± 0.4, (6.4–9.8)
Albumin (g/dL)	4.5 ± 0.6, (3.2–4.9)
Urea (mg/dL)	34.9 ± 7.4, (18–51)
Adherence to Mediterranean diet (PREDIMED)	
Low, medium, high (%)	15.7 / 74.2 / 10.1
IPAQ score	
Low, medium, high (%)	61.1 / 32.2 / 6.7

[a]Values are shown as mean ± SD and range, or percentage. [b]Low refers to ≤10 g of alcohol consumption per day [24]. [c]oxLDLc/LDLc and oxLDL/ApoB ratios (dimensionless) have been multiplied by 10^6 for readability. PREDIMED adherence categories were calculated from the 14-item Mediterranean diet questionnaire (score range 0–14) and classified as low (≤5), medium (6–9), and high (≥10) [25]. IPAQ categories were derived from the International Physical Activity Questionnaire-Short Form [26], and were

normalised to a 0–100 scale for readability and classified as low (0–33), moderate (34–66), and high (67–100) physical activity levels

Table S3 Distribution of metabotypes and their clusters (MCs) in postmenopausal women at inclusion and after consuming polyphenols (PPs) for 8 weeks

	**Baseline**	**After PP intake**
**Individual metabotypes:**		
UMA, UMB, UM0	71.9%, 28.1%, 0% (n=64, n=25, n=0)	63%, 37%, 0% (n=49, n=29, n=0)
EP, ENP	50.6%, 49.4% (n=45, n=44)	59%, 41% (n=46, n=32)
LP, LNP	50.6%, 49.4% (n=45, n=44)	56%, 44% (n=44, n=34)
**Metabotype clusters (MCs):**		
**MC1:** UMB+ENP+LP	10.1% (n=9)	12.8% (n=10)
**MC2:** UMA+ENP+LP	15.7% (n=14)	9.0% (n=7)
**MC3:** UMA+EP+LP	15.7% (n=14)	20.5% (n=16)
**MC4:** UMB+EP+LP	9.0% (n=8)	14.1% (n=11)
**MC5:** UMA+ENP+LNP	18.0% (n=16)	14.1% (n=11)
**MC6:** UMB+ENP+LNP	5.6% (n=5)	5.1% (n=4)
**MC7:** UMA+EP+LNP	22.5% (n=20)	19.2% (n=15)
**MC8:** UMB+EP+LNP	3.4% (n=3)	5.1% (n=4)
**MC9:** UM0+EP+LNP	0%	0%
**MC10:** UM0+EP+LNP	0%	0%
**MC11:** UM0+EP+LP	0%	0%
**MC12:** UM0+ENP+LP	0%	0%

Values are shown as percentages (adapted from Jarrín-Orozco et al. (2025)). The MC numbering follows that previously used by Iglesias-Aguirre et al. (2022). UMA, urolithin A metabotype; UMB, urolithin B metabotype; UM0, urolithin non-producer; EP, equol producer; ENP, equol non-producer; LP, lunularin producer; LNP, lunularin non-producer.

Table S4 Metabotype-stratified analysis of changes in blood lipids (TChol, LDLc, TGs)

Stratification level	Number of subgroups evaluated	Variables assessed	Significance
Individual metabotypes	6 (UMA, UMB, EP, ENP, LP, LNP)	TChol, LDLc, TGs	Baseline vs PPs: $p < 0.001$ Baseline vs Pla: $p > 0.05$
Metabotype clusters (MCs)	8 (MC1, MC2, MC3, MC4, MC5, MC6, MC7, MC8)	TChol, LDLc, TGs	Baseline vs PPs: $p < 0.001$ Baseline vs Pla: $p > 0.05$

Statistical analysis performed using a two-way repeated-measures ANOVA. Lipids increased significantly in all metabotypes and MCs after PPs, indicating the absence of a metabotype-dependent effect. PPs, polyphenol intervention. UMA, urolithin A metabotype; UMB, urolithin B metabotype; UM0, urolithin non-producer; EP, equol producer; ENP, equol non-producer; LP, lunularin producer; LNP, lunularin non-producer. The MC numbering follows that previously used by Iglesias-Aguirre et al. 2022.

Table S5. Covariate analysis of metabotype-stratified changes in blood lipids

Stratification level	Number of subgroups evaluated	Variables assessed	Covariates assessed	r range	Correlation *p*-value range
Individual metabotypes	6 (UMA, UMB, EP, ENP, LP, LNP)	TChol, LDLc, TGs	BMI, age at menopause, years since menopause, Mediterranean diet adherence, physical activity	-0.22 to 0.28	0.156 to 0.876
Metabotype clusters (MCs)	8 (MC1, MC2, MC3, MC4, MC5, MC6, MC7, MC8)	TChol, LDLc, TGs	BMI, age at menopause, years since menopause, Mediterranean diet adherence, physical activity	-0.29 to 0.25	0.112 to 0.921

No covariate showed a significant association with changes in lipids. Covariate influence was evaluated using Pearson/Spearman correlations and exploratory multiple linear regression models. UMA, urolithin A metabotype; UMB, urolithin B metabotype; UM0, urolithin non-producer; EP, equol producer; ENP, equol non-producer; LP, lunularin producer; LNP, lunularin non-producer. The MC numbering follows that previously used by Iglesias-Aguirre et al. 2022.

Table S6. Metabotype-stratified statistical analysis of oxLDL-related ratios.

Stratification level	Number of subgroups evaluated	Variables assessed	Significance
Individual metabolite amounts	Uro-A, Uro-B, IsoUro-A, equol, LUNU and derived phase-II metabolites	ApoB, LDLc/ApoB, oxLDL/LDLc, ox-LDL/ApoB	No significant associations; $p > 0.05$
Individual metabotypes	6 (UMA, UMB, EP, ENP, LP, LNP)	ApoB, LDLc/ApoB, oxLDL/LDLc, ox-LDL/ApoB	Baseline vs PPs: $p < 0.001$ Baseline vs Pla: $p > 0.05$
Metabotype clusters (MCs)	8 (MC1, MC2, MC3, MC4, MC5, MC6, MC7, MC8)	ApoB, LDLc/ApoB, oxLDL/LDLc, ox-LDL/ApoB	Baseline vs PPs: $p < 0.001$ Baseline vs Pla: $p > 0.05$

Statistical analysis performed using a two-way repeated-measures ANOVA. PPs, polyphenol intervention. UMA, urolithin A metabotype; UMB, urolithin B metabotype; UM0, urolithin non-producer; EP, equol producer; ENP, equol non-producer; LP, lunularin producer; LNP, lunularin non-producer. The MC numbering follows that previously used by Iglesias-Aguirre et al. 2022.

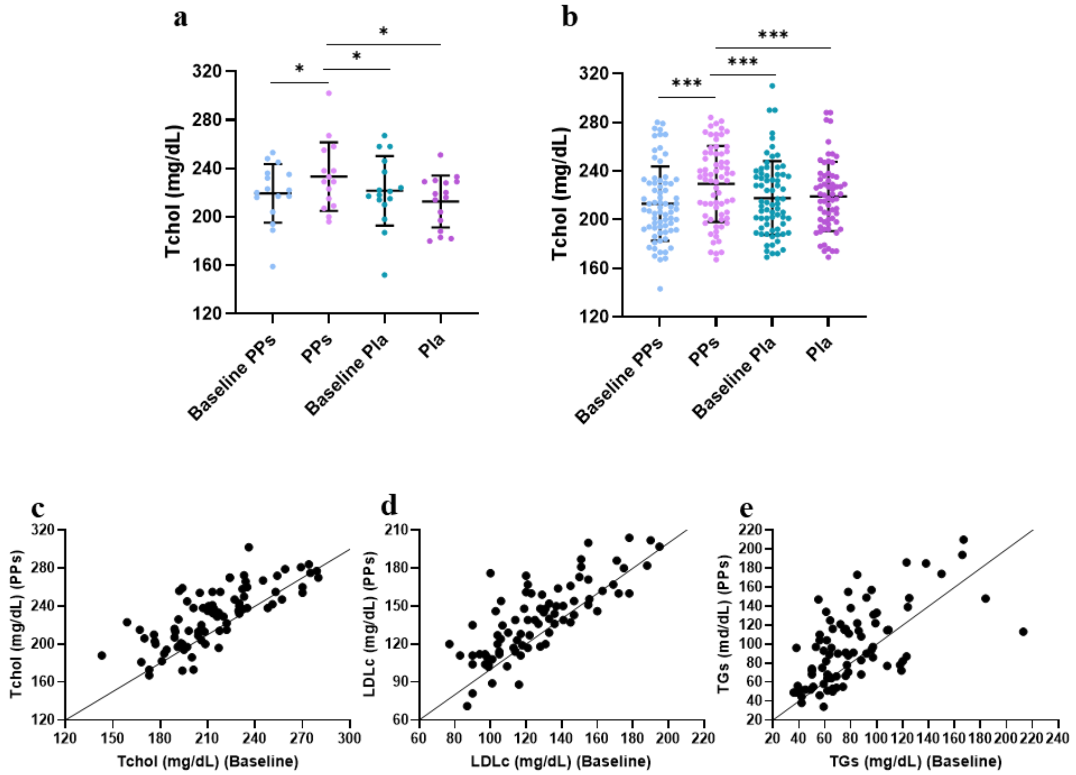

Fig. S1 Changes in Tchol after polyphenol or placebo consumption in Madrid (**a**) and Murcia (**b**) **p*<0.05; *** *p*<0.001. PPs, polyphenol-rich plant mixture; Pla, placebo. Similar trends and statistical significance were observed for LDLc and TGs (data not shown). Panels (**c**), (**d**), and (**e**) display baseline versus post-intervention levels of Tchol, LDLc, and TGs, respectively, following PPs supplementation

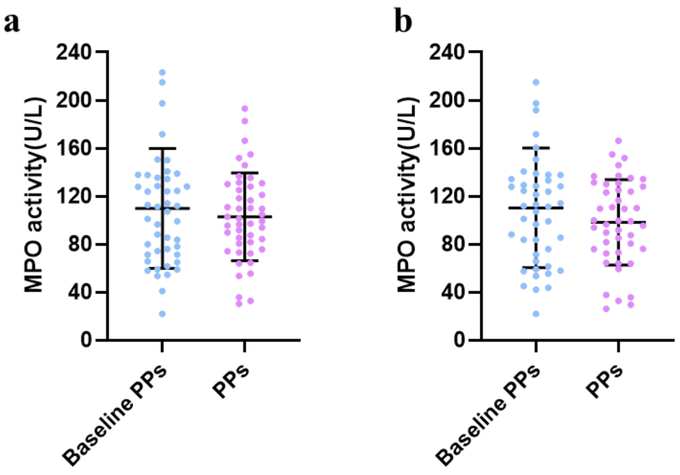

Fig. S2 Change in MPO activity at baseline and after consuming PPs. (**a**) EP, equol producers (*p* = 0.762), (**b**) UMA, urolithin A metabotype (*p* = 0.286).

References

Jarrín-Orozco MP, Romo-Vaquero M, Carrascosa C et al. (2025) Polyphenol-Related Gut Metabotype Signatures Linked to Quality of Life in Postmenopausal Women: A Randomized, Placebo-Controlled Crossover Trial. Nutrients. 17:3572. https://doi.org/10.3390/nu17223572

Jarrín-Orozco MP, García-Nicolás M, Romo-Vaquero M et al (2026) Precision health targeting TMAO in postmenopausal women: polyphenol effects modulated by urolithin A and equol metabotypes in a randomised, placebo-controlled crossover trial. Food Funct. 17:2037–2049. https://doi.org/10.1039/d5fo05461c

Iglesias-Aguirre CE, Vallejo F, Beltrán D et al (2022) Lunularin producers versus non-producers: novel human metabotypes associated with the metabolism of resveratrol by the gut microbiota. J Agric Food Chem 70:10521–10531. https://doi.org/10.1021/acs.jafc.2c04518
